# Evaluation of outcomes in patients with diabetic foot ulcers based on initial assessment at admission

**DOI:** 10.12669/pjms.42.8.15773

**Published:** 2026-08

**Authors:** Sadaqat Ali, Tahir Ghaffar, Syed Hilal Badshah

**Affiliations:** 1 Sadaqat Ali, FCPS Medicine, Department of Endocrinology and Diabetes Mellitus, Hayatabad Medical Complex, Peshawar, Pakistan; 2 Tahir Ghaffar, FCPS Medicine, FCPS Endocrinology, MRCP, Department of Endocrinology and Diabetes Mellitus, Hayatabad Medical Complex, Peshawar, Pakistan; 3 Syed Hilal Badshah, FCPS Medicine, Department of Endocrinology and Diabetes Mellitus, Hayatabad Medical Complex, Peshawar, Pakistan

**Keywords:** Amputation, DFU, Glycemic control, HbA1c, Infection severity, Peripheral arterial disease (PAD), Risk factors, Wagner classification

## Abstract

**Objective:**

To determine the outcomes of diabetic foot ulcers in hospitalized patients based on initial evaluation.

**Methodology:**

A cross-sectional study was conducted in the Endocrinology department, Hayatabad Medical Complex Peshawar, Pakistan, from June 26, 2025 to December 26, 2025. Consecutive sampling was used to cover 108 individuals who had been admitted with DFU. Clinical, laboratory, and demographic information was gathered, including the duration of diabetes, Wagner grade, peripheral pulses, HbA1c, total leukocyte count (TLC), X-ray foot findings and vascular assessment. Clinical outcomes were divided into four categories: below-knee amputation (BKA), forefoot/ mid foot amputation, toe amputation, and conservative care. Data were analyzed by using Chi-square tests and multinomial logistic regression (p ≤ 0.05).

**Results:**

Most patients were male (63%) and aged 41–70 years (85.2%). Diabetes duration >10 years was present in 63.9% and poor glycemic control (HbA1c >7.5%) was observed in 90.8% of patients. Wagner grade and X-ray evidence of osteomyelitis was significantly associated with poorer outcomes (p <0.001 and p=0.027 respectively). Multinomial regression identified Wagner grade had the strongest association with the amputation (BKA: OR 6.50; TOE: OR 3.12). X-ray findings were independently related to toe amputation (OR 0.33).

**Conclusion:**

Initial ulcer severity and bone involvement on X-ray strongly predict amputation in hospitalized patients. Timely identification and aggressive treatment of severe ulcers may reduce limb loss.

## INTRODUCTION

The most common cause of preventable limb loss in the world is diabetes mellitus. High incidence of mortality and morbidity is found in patients with diabetic foot ulcer (DFU).^1^ DFUs affect about 18.6 million individuals worldwide, with 1.6 million cases in the United States each year. The management of DFUs places a substantial economic burden on healthcare systems.^2^ It has a prevalence of 6.3% worldwide, often causing a high amputation rate.^3^ There is a 25% risk of developing foot ulcer in diabetic patients and mortality rate linked with DFU is about 5% in the first year and 42% within five years.^4^,^5^ The etiology can be classified into three types i.e. neuropathic (35%),ischemic (15%), and mixed neuroischemic (50%).^6^ A prospective cohort study at the Baqai Institute of Diabetology and Endocrinology in Karachi, revealed that 9.6% of patients with diabetic foot ulcers had major amputations, while 44.6% had minor amputations. Almost half of the patients (45.6%) recovered without requiring amputation. These results demonstrate the major burden of limb loss correlated with diabetic foot complications within the local population.^7^

A meta-analysis has identified multiple risk factors for the occurrence of DFUs. High blood glucose level, HbA1c, triglycerides, CRP, and decreased ankle-brachial index (ABI) and total protein levels were significantly increased risk of DFUs. Additionally, diabetes related complications including nephropathy, neuropathy, retinopathy, and high systolic blood pressure (SBP) further contribute to the ulcer development. Other factors such as longer duration of diabetes, increased body mass index (BMI) and male gender have also been identified as important risk factor.^8^ Recent evidence suggests the annual incidence of DFU is about 2% among patients with diabetes, while up to 34% of those with Type-2 diabetes, experience a DFU at least once in their lifetime. Factors such as infections, Peripheral arterial disease (PAD) and ulcer severity play important roles in the prognosis and management.^9^

Current research emphasizes the importance of timely detection and multidisciplinary treatment to improve DFU outcomes. Prognosis is heavily influenced by infection severity, ischemia extent, and ulceration depth. The absence of standardized evaluation methods and variability in clinical practices hinder the routine identification of high-risk individuals.

The importance of this research is to identify DFU outcomes using the parameters of the initial assessment, identify essential determinants of healing, amputation, and mortality, and contribute to the development of risk stratification tools and targeted interventions. These results will improve clinical decision-making, improve patient outcomes and contribute to the advancing body of evidence in diabetic foot care.

## METHODOLOGY

This descriptive cross-sectional study was carried out in the Department of Endocrinology at Hayatabad Medical Complex (HMC), Peshawar, Pakistan, over a minimum period of six months, from June 26 to December 26, 2025. The sample size was calculated using the WHO sample size calculator (version 1.1), assuming a 95% confidence level, an anticipated population proportion of approximately 10%, and an absolute precision of 7%. However, to increase the power and reliability of the study, a total of 108 patients were included. The anticipated proportion was based on previously published literature^7^

*Inclusion criteria:* Patients were recruited using non-probability consecutive sampling. Hospitalized patients of all ages and both genders with a confirmed case of DFU were considered for inclusion.

### Exclusion criteria:

Patients with non-diabetic foot ulcers were excluded from the study. Additionally, patients who left the hospital against medical advice before completion of the initial assessment were not included in the final analysis.

### Ethical Approval:

It was obtained from the hospital ethical review committee with Ref. No.2726; Dated: June 26, 2025.

### Data Collection Procedure and Analysis:

Participants who met the criteria were included in the study. The study purpose and potential benefits were explained and written informed consent was obtained. Demographic characteristics were recorded in the Proforma. Routine examination was conducted after a thorough history and clinical examination. The lower limbs were examined neurovascularly. The nature and severity of foot lesions were identified. Patients with absent distal pulses on clinical examination were considered to have clinical evidence of peripheral ischemia and neuropathy was assessed by using a 128-Hz of tuning fork to evaluate vibration perception. Metabolic markers and imaging investigations were evaluated such as blood glucose, lipid profile, TLC, HbA1c, X-ray foot and outcomes. A fundus examination detected Retinopathy. The participant’s age, gender, duration of diabetes and atherosclerosis risk factors (smoking, hypertension, and hyperlipidemia) were all taken into account. A pre-designed proforma was used for standardized data collection, and strict inclusion and exclusion criteria were applied to minimize potential bias.

### Statistical analysis:

All collected data were analyzed with the Statistical Package for the social sciences (SPSS) version 20.00. Quantitative variables were summarized as Mean and SD, While qualitative variables were expressed as frequencies and percentage. The chi-square test was used to determine the statistical associations. The p-value of ≤0.05 was considered statistically significant. All of the findings were displayed in the form of tables, graphs, or charts.

## RESULTS

The study included 108 patients with diabetic foot ulcers. Male patients were more common, accounting for 63.0% (n = 68) of cases, while females made up 37.0% (n = 40). Most patients were aged 41–70 years (85.2%), whereas 9.3% were older than 70 years and only 5.6% were below 40 years of age. All of them had Type-2 diabetes mellitus. Patients with diabetes for more than 10 years accounted for 63.9% (n = 69) of the study population, and this group showed a higher frequency of surgical interventions, including toe (TOE) and below-knee amputations (BKA), compared to those who had disease lasted less than five years (15.7%, n = 17) or 5–10 years (20.4%, n = 22). Despite these findings, there was no statistically significant association between the duration of diabetes and ulcer outcome (p = 0.086).

The majority of patients had poor glycemic control with 49.1% (n = 53) having HbA1c levels between 7.5–11% and 41.7% (n = 45) exceeding 11%, while only 9.3% (n = 10) had HbA1c below 7.5%. Although higher HbA1c levels were observed among patients undergoing amputation, the association between HbA1c and outcome was not statistically significant (p=0.926). Similarly, elevated total leukocyte counts were more commonly observed in patients undergoing amputation, particularly among those with TLC greater than 20,000 cells/mm³ (19.4%, n = 21), while 49.1% (n = 53) had counts between 11,000–20,000 and 31.5% (n = 34) had counts below 11,000. Although higher TLC values suggested more severe infection, this variable did not reach statistical significance in relation to ulcer outcome (p = 0.076). Ischemia, assessed by the absence of peripheral pulses, was present in 17.6% (n = 19) of patients and was linked to a higher risk TOE or BKA, reflecting poor limb perfusion however, no significant association was discovered between ischemia and outcome (p = 0.552). Wound severity, measured by Wagner grade, was strongly linked to the results of DFU (Chi-square = 43.36, p = 0.00002).

**Table-I:** Baseline characteristics on initial Assessment with their frequencies, percentages and P-values.

Variables	Frequencies	Percentages	P-values
***Gender***			0.594
Male	68	63%	
Female	40	37%	
***Age***			0.295
Less than 40 years	06	5.6%	
41---------70 years	92	85.2%	
Greater than 70 years	10	9.3%	
Type of diabetes mellitus	108	100	
***Duration of DM***			0.086
< 5 years	17	15.7%	
5---10 years	22	20.4%	
> 10 years	69	63.9%	
***Smoking***			0.746
Yes	19	17.6	
No	89	82.4	
***Diabetic foot ulcer***			0.438
Single ulcer	65	60.2	
Multiple ulcer	43	39.8	
***Peripheral Pulse***			0.552
Present	89	82.4	
Absent	19	17.6	
***Glycemic Control (HBA1c)***		
<7.5%	10	9.3
7.5-11%	53	49.1
>11%	45	41.7
***Total Leucocyte Count***			0.076
<11,000	34	31.5	
11,000-20,000	53	49.1	
>20,000	21	19.4	
***X-Ray findings of foot***			0.000
Present (osteomyelitis)	54	50%	
Absent	54	50%	
***Common Comorbidities***			0.715
IHD	09	8.3	
CKD	22	20.4	
Retinopathy	40	37	

Ulcers of Grade-1 and 2 were mostly treated without surgery. Higher grades, from three to five, more often needed toe or below-knee amputation. This shows that more severe ulcers are more likely to require surgery. X-ray evidence of osteomyelitis was seen in 50% of patients. It was also significantly associated with the treatment outcome (p = 0.000). Patients with bone involvement on X-ray were more likely to undergo surgical procedures. Among comorbidities, diabetic retinopathy was most common (37%), followed by chronic kidney disease (20.4%) and ischemic heart disease (8.3%). The multinomial logistic regression model significantly improved prediction of DFU outcomes (p < 0.001) and demonstrated excellent fit, with wound grade identified as the strongest independent predictor, followed by foot X-ray findings, while other clinical and laboratory variables were not significant. Higher wound grade markedly increased the odds of below-knee and toe amputation, and abnormal X-ray findings were independently associated with toe amputation.

## DISCUSSIONS

This study of 108 hospitalized patients with diabetic foot ulcers (DFUs) indicates that initial clinical and laboratory assessment can predict ulcer outcomes, with ulcer severity and radiographic evidence of bone involvement being the strongest determinants of amputation. The majority of patients were male and belonged to middle and older age groups. Similar demographic patterns have been reported in recent hospital-based studies of diabetic foot disease, where male predominance and increasing age are commonly observed among admitted patients.^10^ A multicenter study from China also reported that men constituted the majority of patients requiring hospital treatment for DFU. In that study, more severe ulcers and the presence of peripheral arterial diseases were major warning sign for amputation, independent of age and sex. These observations are comparable to our findings and support the role of demographic and clinical severity factors in determining outcomes of diabetic foot ulcers.^11^

Poor glycemic control, as reflected by elevated HbA1c in the majority of patients, was more common in those undergoing surgical intervention, but it did not show statistical significance for outcome in multivariable analysis. This is consistent with recent cohort studies that found HbA1c to be a marker of chronic metabolic burden rather than a *direct* predictor of short-term surgical outcome, contrasting with older studies that emphasized glycemic control but without adjustment for ulcer severity or local infection status.^12^

**Table-II:** Statistical Analysis of Factors Associated with DFU Outcome.

Variables	BKA	Toe	Forefoot/Midfoot	Conservatively Management	P-value
***Duration of DM***					0.086
<5years	01	09	00	07	
5-10 years	04	05	03	10	
>10 years	10	25	01	33	
***DFU***					0.438
Single ulcer	10	26	03	26	
Multiple ulcer	05	13	01	24	
***HBA1c (HBA1c)***					0.926
<7.5%	1	5	00	04	
7.5-11%	9	18	02	24	
>11%	5	16	02	22	
***Peripheral Pulse***					0.552
Present	12	30	03	44	
Absent	03	09	01	06	
***Wound Grading***					0.000
G 1	00	00	00	03	
G 2	00	00	00	20	
G 3	04	16	01	15	
G 4	05	16	02	09	
G 5	06	07	01	02	
***TLC at initial***					0.076
<11,000	05	20	02	26	
11,000-20,000	07	03	01	10	
>20,000	03	16	01	14	

In our study, TLC was higher among patients requiring surgical intervention, especially BKA, suggesting more severe infection. However, this variable did not achieve statistical significance in either univariate or multivariate analysis. Although in some other studies, infection and poor blood flow are known to increase the risk of amputation.^13^Absent peripheral pulses were identified in 17.6% of patients and were associated with a higher frequency of amputations, although the relationship was not statistically significant. This contrasts with large cohort studies from Western populations, where ischemia is a strong predictor of major amputation.^14^ The relatively lower prevalence of clinically evident ischemia in this study and the lack of objective vascular measurement such as ankle-brachial indices or Doppler investigation) may explain this discrepancy.

Wound severity, evaluated by the Wagner grading system, was the strongest factor influencing the outcome in both univariate and multinomial regression analyses. Participants with greater Wagner values were more prone to undergo toe or below-knee amputation, while those with grade one or two ulcers were mostly treated without surgery. Similarly, a 2024 multi-center Chinese cohort found that Wagner grades ≥3 were independently linked to an elevated risk of amputation among individuals with DFU. In addition, half of the patient’s showed osteomyelitis on X-ray, and this was an independent predictor of treatment outcome. In the detailed analysis, abnormal X-ray findings were significantly associated with toe amputation, indicating that bone involvement increases the chance of surgical management.^14^ These findings agree with studies showing that osteomyelitis often requires surgery even when antibiotics are used correctly. The study revealed that severe ulcers combined with osteomyelitis increases the risk of amputation, highlighting the need for early detection and management.

During our study, an overall rate of amputation (50% when minor and major procedures are combined) was higher than the pooled amputation rate of ~31% reported in a recent meta-analysis of diabetic foot ulcer cohorts, which ranged from 14% to 59% across settings. This analysis found that roughly one-third of patients with DFUs eventually require lower extremity amputation, with male sex and comorbidities increasing risk.^15^ This suggests that our hospitalized population may have had more severe disease or delayed presentation. Some hospital-based studies have shown even higher rates. For example, an observational cohort at a major medical center reported an amputation rate of 44% during the initial admission for patients with DFUs, with most amputations involving toes or feet.^16^

### Limitations:

First, the cross-sectional study has constraints in establishing direct correlation between baseline characteristics and subsequent diabetic foot ulcer outcomes. Second, the study took place at a single specialized hospital, which may have restricted the results are generalizable to other medical facilities. Third, the limited number of participants may have reduced the statistical power to identify insignificant relationships, such as the independent role of HbA1c. Furthermore, follow-up data on ulcer healing period, recurrence and Long-term mortality was not studied.

### Strengths:

Despite these limitations, the study has notable strengths, as it demonstrates that initial bedside assessment with radiographic evaluation significantly impact outcome. Higher Wagner grades and X-ray evidence of osteomyelitis were the strongest associated factors of amputation, although glycemic control and routine laboratory indicators have poor predictive value. Early detection of serious ulceration and bone involvement is crucial for reducing the risk of limb loss. This requires multidisciplinary treatment and surgical intervention.

## CONCLUSION

Initial assessment at hospital admission provides valuable prognostic information in patients with diabetic foot ulcers. Higher Wagner grades and radiographic evidence of osteomyelitis were independent predictors of amputation, whereas diabetes duration, HbA1c, total leukocyte count, and peripheral pulse status were not independently associated with clinical outcome. Early recognition of severe ulceration and bone involvement may facilitate timely multidisciplinary intervention and potentially reduce limb loss.

### Recommendations:

Future research should include large multicenter prospective studies across different healthcare settings to improve the generalizability of findings. Adding more detailed assessments such as objective vascular evaluation, microbiological profiling, inflammatory markers, ulcer healing time, recurrence, and long-term outcomes would help provide a better understanding of how diabetic foot ulcers progress over time. In addition, developing and validating simple, practical risk stratification tools may support earlier identification of high-risk patients and facilitate timely multidisciplinary care.

### Authors’ Contribution: SA:

Data collection, data analysis, literature review and interpretation of data, performed statistical analysis & drafted the manuscript. **TG:** Conceived, designed, and critically revised the manuscript. **SHB:** Participated in analysis and literature review. All authors provided final approval for publication of the manuscript and are responsible for the integrity of the study.
